# Adopting and implementing nutrition guidelines in recreational facilities: Public and private sector roles. A multiple case study

**DOI:** 10.1186/1471-2458-12-376

**Published:** 2012-05-25

**Authors:** Dana Lee Olstad, Kim D Raine, Linda J McCargar

**Affiliations:** 1 Alberta Institute for Human Nutrition, 4-126 Li Ka Shing Centre, 8606 112 St, University of Alberta, Edmonton, AB T6G 2E1, Canada; 2 Department of Agricultural, Food and Nutritional Science, 4–10 Agriculture/Forestry Centre, University of Alberta, Edmonton, AB T6G 2P5, Canada; 3 Centre for Health Promotion Studies, 3–300 ECHA, 11405 87 Ave, University of Alberta, Edmonton, AB T6G 1C9, Canada

**Keywords:** Childhood obesity, Food environment, Recreational facility, Diffusion of Innovations, Mixed methods, Case study, Nutrition guidelines, Adoption, Implementation

## Abstract

**Background:**

Recreational facilities are an important community resource for health promotion because they provide access to affordable physical activities. However, despite their health mandate, many have unhealthy food environments that may paradoxically increase the risk of childhood obesity. The Alberta Nutrition Guidelines for Children and Youth (ANGCY) are government-initiated, voluntary guidelines intended to facilitate children’s access to healthy food and beverage choices in schools, childcare and recreational facilities, however few recreational facilities are using them.

**Methods:**

We used mixed methods within an exploratory multiple case study to examine factors that influenced adoption and implementation of the ANGCY and the nature of the food environment within three cases: an adopter, a semi-adopter and a non-adopter of the ANGCY. Diffusion of Innovations theory provided the theoretical platform for the study. Qualitative data were generated through interviews, observations, and document reviews, and were analysed using directed content analysis. Set theoretic logic was used to identify factors that differentiated adopters from the non-adopter. Quantitative sales data were also collected, and the quality of the food environment was scored using four complementary tools.

**Results:**

The keys to adoption and implementation of nutrition guidelines in recreational facilities related to the managers’ nutrition-related knowledge, beliefs and perceptions, as these shaped his decisions and actions. The manager, however, could not accomplish adoption and implementation alone. Intersectoral linkages with schools and formal, health promoting partnerships with industry were also important for adoption and implementation to occur. The food environment in facilities that had adopted the ANGCY did not appear to be superior to the food environment in facilities that had not adopted the ANGCY.

**Conclusions:**

ANGCY uptake may continue to falter under the current voluntary approach, as the environmental supports for voluntary action are poor. Where ANGCY uptake does occur, changes to the food environment may be relatively minor. Stronger government measures may be needed to require recreational facilities to improve their food environments and to limit availability of unhealthy foods.

## Background

Historically, obesity prevalence was low and relatively unchanging among children, however, in nations that regularly monitored population level height and weight statistics, an upward trend in the prevalence of childhood obesity emerged in the 1970s and 1980s [1]. Although recent data suggest it may now have slowed or even plateaued in some nations [2], the continued high prevalence of obesity threatens to reduce the life expectancy of the current generation of children below that of previous generations [3].

The causes of overweight and obesity are multifactorial. A socio-environmental paradigm provides a framework for understanding obesity as a consequence of the complex and dynamic interplay between individuals (including biological and behavioural factors) and their environments. Children may be particularly vulnerable to obesity-promoting environmental influences, given that they have little autonomy and adults determine the content of their environments. Empirical evidence now confirms that social, physical, economic [4-6], and political aspects [7,8] of children’s food environments influence their dietary behaviors and body weights. Policy has proven to be a powerful means of shaping the environmental conditions that affect health [9,10], and is therefore increasingly being used as a strategy to reduce children’s exposure to unhealthy, obesity-promoting food environments.

Progress in using policy to reduce children’s exposure to unhealthy food environments in schools [8,11] has generated interest in using similar strategies to improve recreational facility food environments, as despite their health mandate, many have unhealthy food environments that may paradoxically increase the risk of childhood obesity [12-17]. Indeed, a recent systematic review found no clear association between body weight and youth sports participation, a finding that may be related to direct access to excess calories in sport settings [18]. Several Canadian cities [19-21] and provinces [15,22-24] have therefore mandated or recommended that recreational facilities adhere to nutrition guidelines. These initiatives have had limited success [21,25], although a recent study showed potential for small positive change when significant support was provided to recreational facilities [15].

In Alberta, Canada, the Alberta Nutrition Guidelines for Children and Youth (ANGCY) are voluntary, government-issued guidelines intended to facilitate children’s access to healthy food and beverage choices within schools, childcare and recreational facilities [22]. Findings suggest that one year following their release, awareness, adoption and implementation of the guidelines was low in recreational facilities [16]. Although some of the factors inhibiting the use of nutrition guidelines in recreational facilities have been identified [14-16], they have not been examined in an in-depth manner. It is also unclear from these studies which factors are the most influential and might be sufficient to dissuade or compel adoption and implementation of nutrition guidelines in various contexts. Therefore, we sought to take advantage of this natural experiment by investigating the factors that facilitated and acted as barriers to adopting and implementing the ANGCY in recreational facilities in an in-depth way. Specifically, we used mixed methods within an exploratory multiple case study to answer the following two questions: 1) What is the nature of the food environment within recreational facilities that have and have not adopted the ANGCY? 2) What factors influenced adoption and implementation of the ANGCY within these recreational facilities? We define adoption as a one-time mental decision to follow the ANGCY, whereas implementation refers to multiple acts that must be repeated over time to put the decision into practice [26].

## Methods

### Study design

#### Theoretical framework

Diffusion is a process whereby an innovation is communicated over time among the members of a social network [26]. It is a social process consisting of interpersonal network exchange and social modeling by adopters to those who are influenced to follow their lead [26]. Diffusion of Innovations can provide a conceptual basis for understanding how and why the ANGCY spread or failed to spread among recreational facilities in Alberta, as, because they are not mandated policy, their adoption is not assured, and given their limited formal dissemination, spread is likely to occur via informal, social means. Whereas classical Diffusion of Innovations theory describes the adoption of simple product-based innovations by individuals [26], Greenhalgh et al’s [27] systems approach models the transfer of complex process-based innovations in organizations (Table 1). The comprehensiveness and utility of the model is attested to by the work of others who have conducted similar reviews and/or who have used the model to structure investigations [28-31].

**Table 1:** Major components of Greenhalgh et al’s conceptual model for considering the determinants of diffusion, dissemination and implementation of innovations in organizations

**Framework components**	**Description**	**Examples**
Attributes of the innovation	Perceived attributes of the innovation explain much of the variance in adoption rates	Relative advantage, complexity, observability
Organizational antecedents for innovation	General features of the organization that make it more or less innovative	Receptive context for change, absorptive capacity
Organizational readiness for innovation	Readiness and/or willingness of the organization to adopt a particular innovation	Power balances, tension for change, innovation-system fit
Adopters and the adoption process	Influential aspects of adopters and of adoption as a process	Meaning of the innovation to potential adopters
Processes of assimilation	Organizations may move back and forth between initiation, development and implementation of the innovation	Complex, non-linear processes
Implementation process	Specific steps involved in putting a decision into practice	Effective management, feedback and monitoring
Communication and influence	Means of spreading the innovation	Champions, diffusion, dissemination
Outer context	External influences on the organization	Socio-political climate, environmental stability
Linkage between developers and users	Connections that facilitate movement of the innovation from developers to users	Effective knowledge transfer from developers to users

Source: Based on a systematic review of empirical research studies [27].

#### Case selection

Potential cases were identified from the results of a randomized provincial telephone survey of publicly funded recreational facilities [16]. Three cases were purposefully chosen based on their conformity to one of three types. An ANGCY full adopter was defined as a facility that had adopted and implemented the ANGCY within its concession(s) and vending machines, while a non-adopter was defined as a facility that had decided not to incorporate ANGCY recommendations into any of its food service operations. A semi-adopter was a facility that was following ANGCY recommendations in its vending machines or in its concession(s), but not both. We hypothesized that a semi-adopter would embody factors that influence adoption and non-adoption within a single case. At the time of the study there was one known full adopter in the province, of a total of approximately 1020 recreational facilities that served food. We were aware that approximately 50 other recreational facilities were offering healthier choices in their vending machines only, although it is not known whether, or to what extent most were using the ANGCY. From these facilities we selected one that was using the ANGCY to a significant extent. A non-adopter was selected based on proximity to the University of Alberta. For simplicity, and consistent with Diffusion of Innovations terminology, we refer to cases in terms of their adoption status as adopters (full adopter and semi-adopter) and the non-adopter. An in-depth case study of the full adopter has been previously published [32].

#### Ethical approval

This study was conducted according to the guidelines laid down in the Declaration of Helsinki and received ethical approval from the Human Research Ethics Board at the University of Alberta. Informants provided written, informed consent prior to participating in this study. To protect the identity of participants, descriptions of the setting are of a general nature and do not include details that might lead to identification of the cases.

### Data generation and analysis

Data generation and analysis were completed concurrently to permit exploration of emerging themes and adjustment of data gathering instruments and procedures. Mixed methods were used for purposes of complementarity and triangulation, while maintaining an overall qualitative drive. When mixed methods are used for the purposes of triangulation and complementarity (ie. component designs), the different methods typically remain independent during data collection and analysis, and are integrated during interpretation [33]. Accordingly, each data source was first analysed independently by a single investigator as described below. A case study database was established to organize and document the chain of evidence, and thorough records of the data gathering and analytical process were also maintained [34,35].

#### Questionnaire

A written questionnaire was sent to each recreational facility manager as the first step in data generation. The questionnaire’s 37 closed and open-ended questions were designed to address discrete aspects of the theoretical framework to identify areas for subsequent qualitative exploration and to collect relevant contextual details. The content of the questionnaire was reviewed by experts in health promotion and nutrition, and by relevant government officials. Quantitative and categorical responses provided by managers were transformed into narrative descriptions for directed content analysis using the coding scheme (described below).

#### Qualitative assessments

##### Interviews

The theoretical framework and insights from questionnaire analysis guided development of a semi-structured interview guide ( Additional file 1). The guide was pilot tested with two managers, and subsequently expanded to ensure that all domains of the framework would be adequately addressed through participation in an interview and completion of a questionnaire. Managers responsible for food service within each recreational facility (n = 5) participated in one to two semi-structured in-person interviews lasting between 50 and 90 minutes. Corroborating evidence and a variety of perspectives were sought by interviewing managers from industry within each facility (n = 7). All interviews were digitally recorded and transcribed verbatim.

##### Observations

Two to three 30 minute observation periods were conducted by two independent observers during different days and times of day at each facility. Observers were guided by a theoretically-informed observation guide to make “grand tour” and “mini-tour” observations [36], the former being more open-ended and the latter more specific to the elements of the theoretical framework. Observations were transcribed. Photos of the food environment were also taken within each facility.

##### Document reviews

A review was conducted of general administrative documents related to each case including policies, food service contracts, goals, objectives and strategic plans. Printed and online sources of municipal statistics were consulted to abstract contextual and organizational variables.

##### Analysis of qualitative data

Directed content analysis is used to validate or conceptually extend a theoretical framework [37] and was therefore highly appropriate in the current study. Using this approach, the theoretical framework guided development of an initial coding and categorizing scheme and operational definitions for the codes [37]. Another member of the research team inspected the coding scheme to ensure congruence with the elements of the theoretical framework. A single investigator applied the identical coding and categorizing scheme to all qualitative data using techniques of memoing, constant comparison and questions. NVivo software (v.9, QSR International, Cambridge, MA) was used to organize the data during analysis.

#### Quantitative assessment of the food environment

##### Food and beverage availability

Food and beverages available within vending machines (items designated as: food, beverages) and concessions (items designated as: main dish items and sides, snacks and desserts, beverages) were recorded and classified according to ANGCY criteria for “choose most often” (consume daily), “choose sometimes” (≤ 3 servings/week), and “choose least often” (≤ 1 serving/week) [22] on the basis of nutrition information obtained from food vendors, package labels, company websites, directly from manufacturers, and where necessary from the Canadian Nutrient File and Food Processor SQL (ESHA Research Inc, Salem, OR). The number of “choose most often”, “choose sometimes” and “choose least often” items available was then expressed as a percentage of the total number of items available for sale.

##### Nutritional profile of vending machine items

Nutrition information for all items within vending machines was obtained from package labels, company websites or directly from the manufacturer. Nutrients present within all vending machine items were then added to derive a total for each type of machine according to shelf space, and an average was derived representing the average nutrient content for a typical item from food and beverage vending machines [38].

##### Nutrition Environment Measures Survey in Restaurants (NEMS-R) assessment

The NEMS-R is a validated observational instrument that provides a comprehensive and quantitative assessment of factors that contribute to food selection in restaurants, including availability of healthy items, barriers and facilitators to healthy eating, pricing, signage and promotions [39]. The same trained researcher completed the NEMS-R in the concession(s) of each facility and determined the NEMS-R score (possible range: -27 to +63) according to the standardized protocols of Saelens et al [39].

##### ANGCY adoption and implementation scores

ANGCY adoption and implementation scores from 0 to 38 were assigned by two raters on the basis of direct observations and review of menus and policies. Each scoring system consists of up to 19 policies or environmental characteristics recommended in the ANGCY (eg. healthier foods should be available, convenient visible) [22], for which facilities received a 0, 1, or 2 according to whether the policy or environmental characteristic was present (1 = partially present, 2 = fully present) or absent (0), with a higher score indicating greater congruence with the ANGCY. Discrepancies between raters were resolved through discussion to arrive at a mutually agreed upon score. Qualitative observations were also recorded for each of the content items. The adoption score indicates whether facilities have formally adopted ANGCY recommendations through developing nutrition policies, while the implementation score provides a quantitative assessment of food environment quality and the degree and fidelity of ANGCY implementation. Development of the scoring systems was informed by the Robert Wood Johnson Foundation’s School Wellness Policy Coding Tool [40]. Researchers and government officials involved in developing the ANGCY assessed the content validity of the scoring systems and judged them to be congruent with ANGCY recommendations. The scoring systems were pilot-tested in one facility by two raters for reliability and to clarify decision rules. The total ANGCY adoption and implementation scores were derived by summing the scores for individual content items. Scores were expressed as a percentage of the total possible score.

##### Data transformation

To facilitate cross-case comparisons of the food environment, for each food environment assessment tool the range of possible scores was divided into quintiles where the top quintile (ie. 81-99%) corresponded to a rating of “very high/healthy”, followed by “healthy/high”, “moderately healthy/high”, “limited” and “very limited”.

#### Sales

Sales data were requested for the concession(s) and vending machines in each facility, however detailed sales data were only provided for non-adopter concessions. Annual sales of “choose most often”, “choose sometimes” and “choose least often” items in those concessions were expressed as a percentage of total sales.

#### Within case report

Following analysis of individual data sources, quantitative and qualitative data were integrated into a single case study data set, and jointly interpreted to produce each case report. Merged data analysis strategies were used, involving side-by-side comparison of qualitative and quantitative data displayed in tables to identify areas of convergence and divergence [41]. Pattern matching [35] was used to explore the fit of the elements of each case with the theoretical framework and to derive propositions for testing during subsequent cases. Member checking [34] strengthened the credibility of findings, whereby feedback from facility managers was obtained and used to verify the facts of each case prior to finalizing each case report. The final case report for each site distilled and synthesized the entire data set into a single, coherent, in-depth narrative and was finalized prior to conducting new cases.

#### Cross-case analysis

The set theoretic approach formed the basis of our cross-case analytic strategy. In contrast to multivariate techniques which assume causal homogeneity, set theoretic logic assumes that there are multiple causal paths to the same outcome, with some factors being sufficient and others necessary [42]. We maintain that this logic is likely to have a greater affinity with the complex causality that characterizes obesity than standard regression models [42].

Case-oriented pattern matching was initially used to produce a narrative synthesis comparing and contrasting findings among cases for each aspect of the theoretical framework. There was a marked degree of congruence in influential factors between the semi-adopter and the full-adopter facilities, and thus they were grouped as adopters where appropriate. Then, in accordance with set theoretic logic and procedures described by Savaya et al [43], areas of convergence and divergence between adopters and non-adopters were identified, explored, and used to finalize the final set of cross-case propositions. Findings were generated via an iterative, abductive cycle, moving back and forth between inductive and deductive reasoning, checking for consistency between emerging patterns and data derived from individual cases, and revising interpretations where indicated.

#### Long-term follow up

Prior to finalizing the multiple case study, managers were contacted to determine whether their ANGCY adoption status had changed, and to obtain information regarding any nutrition-related changes that had been made subsequent to each case study.

## Results

### Context

The multiple case study was conducted in the province of Alberta, Canada. Recreational facilities are ubiquitous throughout urban and rural Alberta. The available infrastructure varies among facilities, but typically includes swimming pools, ice arenas, soccer centres, curling rinks and/or gymnasiums. Approximately 80% of publicly funded recreational facilities in Alberta sell food as a means of generating additional revenue, most commonly through vending machines, however many facilities also contain publicly or privately operated concessions. Features of the recreational facilities included in this study are presented in Table 2. The adopter and semi-adopter cases were each limited to a single facility, however the non-adopter case included observations from four small recreational facilities where food services were managed by a single manager.

**Table 2:** Summary of cases

**Case**	**Full adopter**	**Semi-adopter**	**Non-adopter**
**Facility type**	Large modern multipurpose facility	Large modern multipurpose facility	Four small aging, single purpose facilities
**Funding**	Publicly funded	Publicly funded	Publicly funded
**Food service management**	General manager	General manager	Dedicated concession services manager
**Food service: concession(s)**	An international franchise that had adopted the ANGCY in schools and in the full adopter facility. Popular for its fries and poutine.	1) An international franchise that had adopted the ANGCY in schools and was willing to adopt them in the semi-adopter facility. The company had a healthy brand image.	4 municipally-operated concessions that were not associated with schools and were not willing to adopt the ANGCY in non-adopter facilities. The study focussed on concessions in 2 facilities:
		2) A small local company that had no school-based operations and was not willing to adopt the ANGCY in the semi-adopter facility. Popular for its fries and poutine.	1) Pool café popular for its sandwiches, wraps, and baked goods.
			2) Arena concession with a fast-food style menu.
**Food service: vending machines**	12 machines serviced by a company that had adopted the ANGCY in schools and in the full adopter facility.	21 machines serviced by a company that had adopted the ANGCY in schools and in the semi-adopter facility.	3 machines serviced by a company that had not adopted the ANGCY in schools or in non-adopter facilities.
**Relationship with schools**	Shared a field with 2 high schools. Students came to the facility at lunch primarily to purchase the unhealthy items they could not purchase on their campuses.	No schools within close proximity.	High school students came to the pool café at lunch, presumably to avoid long line-ups and because they preferred the café-style menu to their school’s cafeteria.
**Clients**	> 50% children^1^	> 50% children^1^	> 50% children^1^

ANGCY: Alberta Nutrition Guidelines for Children and Youth.

^1^Children are defined as < 18 years of age.

### Food environment quality

There were differences in the quality of items present within vending machines among facilities that had and had not adopted the ANGCY (Table 3). Compared to non-adopters, facilities that had adopted the ANGCY in vending machines had higher ANGCY implementation scores for food vending machines, greater availability of “choose most often” items, and their vending machine items contained fewer calories on average. Notably, all food vending machines contained few “choose most often” items, as few were available that could be sold in unrefrigerated vending machines. Nevertheless, the semi-adopter attempted to provide healthier choices in food vending machines by filling them primarily with “choose sometimes” items (77% of food items were “choose sometimes”). Beverage vending machines scored better than food machines on all measures. Comments from managers revealed why this was the case, as bottled water was a top selling item, and therefore it was in the financial interests of food vendors to place this healthy item in machines.

**Table 3:** Subjective and objective assessments of vending machine items

**Case**	**Full adopter**	**Semi-adopter**	**Non-adopter**
**Adoption status**	Adopter in vending machines	Adopter in vending machines	Non-adopter in vending machines
***Food vending machines***			
**ANGCY implementation score**	67% High	71% High	41% Moderate
**Availability of CMO food items**	2% Very limited	4%^1^ Very limited	0% None
**Nutrient content of food machine items**	216 kcals, 42% fat, 54% CHO (13g sugar, 1g fibre), 6% protein, 198 mg sodium	155 kcals, 29% fat, 62% CHO (6g sugar, 2g fibre), 8% protein, 218 mg sodium	285 kcals, 35% fat, 60% CHO (22g sugar, 2g fibre), 3% protein, 277 mg sodium
***Beverage vending machines***			
**ANGCY implementation score**	85% Very high	85% Very high	83% Very high
**Availability of CMO beverages**	31% Limited	26% Limited	13% Very limited
**Nutrient content of beverage machine items**	126 kcals, 0% fat, 98% CHO (28g sugar, 0g fibre), 3% protein, 77 mg sodium	107 kcals, 0% fat, 100% CHO (28g sugar, 0g fibre), 0% protein, 130 mg sodium	138 kcals, 0% fat, 100% CHO (38g sugar, 0g fibre), 0% protein, 126 mg sodium
**Managers’ perceptions of the health of vending machine items**	“Our requirement is [that] 25% [of vending items be healthy] and they meet that, but it doesn’t move so it sits there and the other [unhealthy] stuff on top moves… I wish there was better options… to have stuff in there that is new and interesting and does sell.”	“In terms of vending, there are healthier choices. I wouldn’t say it’s successful… [but] it’s better than it was. .. Am I jumping up and down saying we did it? No, because there’s more to do.”	“I’ve actually never really told them what to put in the vending machines. I don’t eat chips, I don’t eat stuff like that, so I don’t even think about it… We did mention to them that we would like some healthy [items]… but other than that… he’s trying to maximize his sales for the stuff that the kids like.”
**Managers’ perception of the proportion of items that are healthy**	25%	25-30%	15%

ANGCY: Alberta Nutrition Guidelines for Children and Youth; CHO: carbohydrate; CMO: choose most often; kcals: calories.

^1^This facility had a much higher proportion of “choose sometimes” food items in vending machines compared to others, at 77% of items. The proportion of “choose sometimes” items in vending machines in other facilities did not exceed 8%.

There were few clear differences in food environment scores between concessions that had and had not adopted the ANGCY (Table 4). The only concession that had adopted the ANGCY scored well in terms of the overall quality of its food environment, with high NEMS-R and ANGCY implementation scores, yet it provided a very limited proportion of healthy items. Most other concessions that had not adopted the ANGCY also scored highly in terms of the quality of their food environments, and had a similarly low proportion of “choose most often” items available, making them virtually indistinguishable from the adopter. An arena concession, a non-adopter with no availability of “choose most often” food items, stood out as having the poorest quality food environment, with consistently low scores on all measures.

**Table 4:** Subjective and objective assessments of the food environment in concessions

**Case**	**Full adopter**	**Semi-adopter**		**Non-adopter**	
**Adoption status**	Franchised concession: adopter	Franchised concession: non-adopter	Local concession: non-adopter	Pool café: non-adopter	Arena concession: non-adopter
**Facility ANGCY adoption score**	82% Very high	0% No formal policies		0% No formal policies	
**ANGCY implementation score**	75% High	66% High	69% High	75% High	47% Moderate
**Availability of CMO items:**					
**Overall**	16% Very limited	22% Limited	11% Very limited	17% Very limited	11% Very limited
***Main dish and side items***	*23%*	*32%*	*14%*	*12%*	*0%*
***Snacks and desserts***	*7%*	*0%*	*0%*	*10%*	*0%*
***Beverages***	*16%*	*20%*	*15%*	*40%*	*24%*
**NEMS-R**^**1**^	+28 Healthy food environment	Healthy food environment^2^	+ 12 Moderately healthy food environment	+ 18 Moderately healthy food environment	+ 3 Limited healthy aspects of food environment
**Managers’ perceptions of the concession food environment**	“My preference would be that we don’t have the poutine and the real unhealthy stuff here… I would like to [have] a different vendor… that doesn’t even have a deep fryer.”	“[We] didn’t have to have a discussion with the [franchised concession]… They don’t have any junk.”	“We think it would be great if they had more of a deli sandwich approach, you know, fresher, more healthy, instead of the focus on the usual high fat [items].”	“I think that what we do is pretty healthy… I mean when we make our muffins and stuff, we always try to make like a healthier option. But then there’s always the… kind of unhealthy option, sort of thing. But they’re both there.”	“We try to use 100% real beef and you know, we try to – we use a healthier oil and just things like that. I mean, I know that it’s still junk food but it’s kind of, it’s the healthier junk food… People always think a hamburger’s not healthy for you but…it’s beef… it’s got lettuce and tomato and cheese on it, you know? So it’s a burger. But it’s still got the protein…”
**Managers’ perception of the proportion of items that are healthy**	Not available	100%	10%	90%	60%

ANGCY: Alberta Nutrition Guidelines for Children and Youth; CMO: choose most often; NEMS-R: Nutrition Environment Measures Survey in Restaurants.

^1^The range of possible scores was −27 to +63.

^2^Given the non-traditional menu in this concession, a modified NEMS-R was completed and the range of possible scores for this facility was −27 to + 51. This facility scored +30 using the modified NEMS-R, which corresponded with the “healthy” quintile on a modified scale.

Interviews and observations provided new dimensions for understanding quantitative findings regarding the quality of the food environment. Agreement was good overall in that managers generally recognized the need to improve the quality of the food environment within their facilities, however in some instances managers perceived they had more healthy options than they actually did. Observations revealed evidence of action on the part of adopter facilities, but also showed just how prominent unhealthy food was within these facilities that claimed to promote healthy lifestyles. Access to food was particularly high in the case of the semi-adopter, with concessions on both floors, and a large number of vending machines throughout the facility. These findings highlight the value of using multiple modes of data collection.

### Sales

Facilities that had implemented the ANGCY perceived that food and beverage sales had fallen as a consequence. In the semi-adopter, the commission they collected on vending machine sales decreased by 16% from 2009 to 2010, and they anticipated a further decline of 14% in 2011. Similarly, in the full adopter facility, annual sales decreased by 17% in the concession from 2009 to 2010 and the vending machine operator estimated that revenues had declined by 20% since implementation began. Data capture systems were too limited to accurately depict the proportion of revenue declines attributable to ANGCY implementation, and which might have been due to other factors such as the economic recession or declines in facility usage. The inability to disentangle the impact of each of these factors was a barrier to greater implementation of the ANGCY, as managers assumed that increasing the proportion of “choose most often” items might further reduce profitability. Notably, annual revenues in two non-adopter concessions declined by 5% and 9%, respectively, over the same period, declines that the manager attributed to reduced facility usage. Table 5 presents industry’s perceptions of food service sales.

**Table 5:** Industry’s perceptions of food service sales

**Managers’ quotations regarding sales**	**Full adopter**	**Semi-adopter**	**Non-adopter**
**Sales of healthy items compared to sales of less healthy items**	“Whether we like it or not they don't want cucumbers with light organic dressing.. What sells is fries and poutine.”	“French fries is what I sell the most.”	“There’s nobody in this business can make money [selling healthy foods].” “If you’re offering the choices they’re always going to go for the unhealthy choice.”
**Perceived impact of the ANGCY on sales**	“It’s devastating… Horrible, our sales have been reduced.”	“Sales dropped 50%.”	“If we went into a high school doing $100,000 a year in sales, you’d be lucky to see $20,000 [if we implemented the ANGCY]. And I’ve done it – in [another province] not here.”

ANGCY: Alberta Nutrition Guidelines for Children and Youth.

Comparison of sales of healthy and unhealthy items was only possible in two non-adopter concessions as others did not provide itemized sales data. In one, a pool café, sales of healthy options closely mirrored their availability, as 17% of menu items available, and 14% of items sold were “choose most often”. In the other, an arena concession, 11% of items available were “choose most often”, while 4% of items sold were “choose most often”. Of the top 15 food and beverage items sold in the pool café, only two were “choose most often” (water, juice), whereas none were “choose most often” in the arena concession. Observations made by researchers and managers in all facilities supported findings of low sales of “choose most often” items, and in particular it was noticed that students from nearby schools came to the full adopter facility at lunch to purchase the unhealthy items they could not purchase on school grounds.

### Impact of factors on adoption and implementation of the ANGCY

#### Factors common across all cases

The comparative analysis was aimed at distinguishing the factors that determined whether or not adoption and implementation occurred, and mirrors the presentation of findings by Savaya et al [43]. First, in Table 6 we detail factors from the theoretical framework that had a similar impact across all cases, acting as barriers, facilitators or neither within all of the facilities. Because they acted in a similar manner across all cases, the barriers in this list were therefore not strong enough to dissuade adoption and implementation, nor were the facilitators strong enough to compel adoption and implementation. We cannot conclude that these factors are not necessary to adoption and implementation, only that their presence, in the case of facilitators, or absence, in the case of barriers, is not sufficient for adoption and implementation to occur. A cause is sufficient if it is invariably (or almost invariably) followed by the outcome, whereas it is necessary if it is present in all instances of the outcome [44].

**Table 6:** **Factors from Greenhalgh et al’s Diffusion of Innovations framework**[27]**that were common across all cases**

**Factor**	**Definition and theoretically predicted impact on adoption and implementation**	**Study findings**	**Influence on adoption and implementation as reported by managers**
***Attributes of the ANGCY***			
**Observability**	If the benefits of the ANGCY are visible to potential adopters they will be adopted more easily [27].	Managers anticipated few visible positive outcomes from adoption: “There’s variety. But a positive outcome, because we have variety, I couldn’t tell you. Like it’s not something that’s a visual thing that I can tell you that I see”. Negative outcomes were expected and were highly visible because sales decreased significantly and many children continued to purchase unhealthy items.	Barrier to adoption and caused adopters to limit the extent of implementation to avoid larger negative financial consequences.
**Task Issues**	Innovations that are relevant to the performance of the user’s work, that improve task performance and are feasible to use are more readily adopted [27].	The recreation sector had not typically incorporated nutritional considerations within its programming and services, and thus managers perceived some incompatibilities between the ANGCY and staff tasks.	Barrier to adoption and implementation.
**Trialability**	Innovations that can be experimented with on a limited basis are more likely to be assimilated [27].	All managers perceived that that they could “test drive” the ANGCY: “I would say we wrote the policy knowing that we would be trying to change it, based on how things went with our contracts. That was sort of the test, I guess, is measuring over the three years whether it was feasible to have them, whether there was public acceptance or backlash.”	Facilitator of adoption.
**Adaptability**	Diffusion research suggests that innovations are not fixed entities and that innovations will be adopted more readily if potential adopters can modify them to suit their own needs [27].	All managers felt free to adapt the ANGCY and recognized that they could implement them to a greater (ie. restrictive format) or lesser extent (ie. choice-based format) to suit their own needs. This perceived flexibility was important as managers attempted to balance competing priorities of a health and financial nature.	Facilitator of adoption and implementation.
**Augmentation**	Innovations are more easily assimilated if training and support are provided to staff [27].	The Alberta government did not provide training nor did recreational facilities train their staff to implement the ANGCY.	No impact on adoption or implementation.
***Organizational antecedents for the ANGCY***			
**Centralization**	Extent to which decision making authority is concentrated or dispersed within an organization [45]. Negatively associated with organizational innovativeness [45].	Centralized decision making was present in all facilities.	Facilitator and barrier to adoption. It was not the hierarchical structure *per se*, but the priorities of those at the top of the hierarchy that mattered.
**Managerial receptivity to change**	Extent to which managers or members of dominant coalition favour change [45]. Positively associated with organizational innovativeness [45].	Adopters regarded the ANGCY as an opportunity for organizational growth. The manager of the non-adopter facilities also demonstrated a strong commitment to change in other areas.	Facilitator of adoption and implementation.
**Slack resources**	An organization’s resources beyond minimal requirements to maintain operations [45]. Positively associated with organizational innovativeness [45].	Managers’ and employees’ time was fully occupied with their primary duties and responsibilities. Managers felt they had no spare resources to commit to ANGCY implementation.	Barrier to adoption and implementation.
***Organizational readiness for the ANGCY***			
**Assessment of implications**	Innovations are more likely to be assimilated if their implications are fully assessed and anticipated [27].	All managers recognized the potential for revenue loss. Adopters selected a choice-based format to limit negative financial repercussions. The non-adopter chose not to adopt the ANGCY for this reason.	Barrier to adoption.
**Resource availability**	Innovations are more likely to be assimilated if there is an adequate and continuing allocation of resources [27,29].	There were few tools available to support implementation. There was limited availability of ANGCY-compliant products in the marketplace.	Barrier to adoption and implementation.
***Linkage***			
**Linkage at the adoption and implementation stage**	Linkage agents can facilitate adoption of innovations by enhancing knowledge exchange between developers and users [46].	The provincial government hired Health Promotion Coordinators were hired to support ANGCY adoption and implementation, however they did not have an influential role in any of the facilities in this study.	No impact on adoption or implementation.
***Outer context***			
**Socio-political context**	The organization’s decision to adopt an innovation and efforts to implement it may be influenced by social norms and prevailing political ideologies.	Managers all believed to varying extents that it was up to individuals to “develop some personal choice skills where they [make] personal choices that are good for them.” While adopters felt their role was “to make sure that [patrons] have those healthy choices in our facilities and hope that they help themselves”, the non-adopter did not think it feasible to make healthy options available in all contexts.	The personal responsibility ethic was a barrier to adoption for the non-adopter and shaped how adopters implemented the ANGCY (ie. it was a barrier to a restrictive format).

ANGCY: Alberta Nutrition Guidelines for Children and Youth.

#### Factors unique to individual cases

Next, we describe factors from the theoretical framework that were influential for adoption and implementation, but were unique to individual cases. These factors may be important for adoption and implementation in particular contexts, and are therefore sufficient, but not necessary for adoption and implementation.

##### Organizational antecedents for the ANGCY

*Formalization:* Adopters contracted out their food service and as a result had to work within the constraints of food vendors whose values differed from their own. The multi-year nature of these contracts also committed them to particular courses of action for several years at a time. Thus, expiration of their three and five year concession and vending machine contracts, respectively, provided much of the initial impetus for adopting the ANGCY in the full adopter facility: “I really think I was motivated solely by the expiration of contracts and it was sort of a do it now or lose [many] years of opportunity… So I was kind of spurred on by the fact that it was kind of now or never.” The manager seized this window of opportunity to develop new vendor contracts that required adherence to the ANGCY.

Conversely, food service contracts were a major barrier to adoption for the semi-adopter, which was nine years into its 20 year food service agreements that allowed food vendors to sell virtually what they liked. Therefore, had its vending machine company not agreed to adopt the ANGCY, the facility would have remained a non-adopter for another 11 years. The degree of formalization was low within non-adopter facilities, as their concession-based food services were publicly delivered by the municipality and industry was not involved. The concession manager felt that the low degree of formalization had not impacted the decision not to adopt the ANGCY.

##### Organizational readiness for the ANGCY

*Power balances****:*** If supporters of adoption are more numerous and strategically placed than opponents, the ANGCY are more likely to be assimilated [27]. The support of powerful persons and organizations proved to be key facilitators of adoption. Within adopter facilities, the support of facility and municipal leaders was a key prerequisite for adoption and implementation of the ANGCY. These individuals determined in what format the ANGCY would be implemented, either one based in choice (where all foods could be sold) or in a restrictive format (where unhealthy foods could not be sold). The support of food vendors was also essential to adoption and implementation. Public sentiment was influential in adopters’ decision to adopt the ANGCY in a choice-based format, but was accorded less importance within the semi-adopter facility.

Encouragement by local School Boards to adopt the ANGCY was an important catalyst for adoption within the full adopter facility. Given the proximity of the recreational facility to two high schools, the manager wanted to support the School Boards’ efforts to adopt the ANGCY by using them as well: “A facilitator also has been the pressure that’s been put on by the School Boards to do different… [they] were leading the charge… and we felt that we needed to support and/or follow that so it wasn’t just them out on a limb…” However, while most stakeholders preferred that the ANGCY be adopted in a choice-based format, School Boards wanted the facility to adopt the ANGCY in a restrictive format similar to their own. Although the facility did not ultimately adopt restrictive policies, continued interaction and dialogue between the recreational facility and the School Boards helped to sustain implementation. Schools were similarly influential in the adoption decision of the semi-adopter, which was strongly encouraged to adopt the ANGCY by a teacher who was also a member of the facility’s governance Board.

Adopters did not experience any overt opposition because adoption was limited in scope. Managers predicted that strong opposition would have emerged had they removed all unhealthy items from the facilities: “There’s no opposition because we [allowed] choice. There’s no threat because they can still sell what they want to sell. Yeah, I’ve felt no opposition.” The support of food vendors was, however, waning in the face of mounting revenue losses. In addition, apathy was a concern for the full adopter, as although his municipality verbally supported implementation, it had not made child health a priority: “I don’t think it ever became a priority for municipal government. So I’m one person, fairly far down the food chain… and so it’s like a fish trying to swim upstream when you’re just one person trying to effect change, you don’t have a whole bunch of time to commit to it, but you want to make some sort of impact… I wish there was someone further up the ladder who was more passionate or interested in [the ANGCY], because then it would probably move…”

The menus of non-adopter concessions reflected the fact that customers, through market forces, held the balance of power within these facilities. The manager was highly sensitive to customer demands and indicated that if customers had asked him to adopt the ANGCY, he would have given serious consideration to doing so. He also did not expect any stakeholders to overtly oppose adoption.

#### Factors that differed between adopters and the non-adopter

Thirdly, we provide an in-depth analysis of the factors that differed between adopters and the non-adopter. To be included in this list, the impact of the factor on adoption and implementation had to be similar in adopters and demonstrate an opposing, or no relationship in the non-adopter. The presence (in the case of facilitators) or absence (in the case of barriers) of these factors was therefore sufficient and may also be necessary for adoption and/or implementation of the ANGCY.

##### Adopters and the adoption process

*Meaning of the ANGCY to managers:* Individuals do not passively receive innovations, instead they engage with them in complex ways before coming to an adoption decision [27]. The adoption process essentially began when the personal values of recreational facility managers regarding the importance of supporting healthy eating in recreational facilities intersected with timely opportunities to do so. For the full adopter, this opportunity came in the form of the near simultaneous expiry of its three and five year food service contracts. For the semi-adopter, a suggestion from a member of the facility’s governance Board provided the initial adoption stimulus. In both cases, managers, energized by their strong personal beliefs, took immediate action. They did not want to lose their window of opportunity to finally align their actions with their beliefs and to truly begin to “walk [their] talk.” Congruence between the ANGCY and the personal philosophies of managers provided a strong foundation for maintenance of the original adoption decision despite the negative financial outcomes that ensued: “If they really want to eat junk food… they can just go to 7–11 and get it because for us, dollars and cents are not - that’s not the motivating factor… [we] believe in what we’re offering and if it’s going to bring in less money, then so be it.”

The non-adopter believed the ANGCY to be a good initiative but saw no need for them, erroneously believing his menu items to be healthy. In addition to the ANGCY having little meaning for this manager, the coincidence of events that encouraged adopters to act on their beliefs was not present in this case.

##### Attributes of the ANGCY

*Complexity:* Practices that are easily understood and communicated are more readily adopted [47]. Managers from adopter facilities described the recommendations and food rating system within the ANGCY as “practical, easy to understand, and user-friendly.” The full adopter appreciated that “the guidelines [spoke] directly to recreation”, and this allowed them to use wording “straight from the guidelines [in their contracts].” This simplicity facilitated policy development within short timelines. By contrast, simplicity was a quality lacking in the guidelines according to the non-adopter, who felt the 103 page ANGCY document was daunting.

*Relative advantage:* Relative advantage is the degree to which managers expect that following the ANGCY will confer advantages over previous practices. If potential adopters do not perceive a relative advantage they will often not consider an innovation further [27]. Managers within recreational facilities had to weigh the potential advantages to be gained from implementing the ANGCY against the negative consequences that might also result.

Food services within adopter facilities were overseen by the facilities’ general managers. These managers had a wide scope of responsibility and placed a high priority on achieving the community wellness aspect of their mandate. They believed the ANGCY could assist them to support wellness. This potential advantage had to be balanced with the possible negative impact of the ANGCY on revenue generation from food services, however, as funding models were often at odds with support for healthy eating: “I think it’s just trying to balance what’s sustainable in terms of support for the facilities because we get revenue or other assets from the sale of [unhealthy foods] at our facilities, and balancing our philosophy and our beliefs in terms of healthy lifestyles… We’re on a teeter-totter… [we] can’t do one without affecting the other one… like you start taking away the revenue and all of a sudden your fees go up and… so now you’ve got kids eating healthy but they’re not going in to swim. We’ve got to balance it somehow.” Therefore, in areas where ANGCY adoption had relatively small negative financial implications (ie. increasing the number of healthy items in concessions and vending machines), adoption proceeded. Conversely, in areas where ANGCY adoption came at a higher financial cost, the relative advantage of adopting the ANGCY was perceived to be low. For this reason, advertising and sponsorship agreements were maintained, and the sale of highly profitable unhealthy items continued (ie. the ANGCY were adopted in a choice-based format), even though managers would have personally preferred a restrictive format. Thus, the ANGCY offered a relative advantage to adopters insofar as they assisted them to achieve their wellness mandate in a financially sustainable manner. One manager found this a particularly troubling reality, and desired a new business model that did not make them dependent upon revenue from the sale of unhealthy foods.

Food services within non-adopter facilities were managed by a concession services manager with a narrow mandate of maintaining profitable food service operations. Thus, support for community wellness was relatively inconsequential for this manager, and he therefore perceived that adopting the ANGCY would put them at a competitive disadvantage. As a consequence, he had little interest in adopting them.

##### Organizational antecedents for the ANGCY

*Professionalism:* Professionalism refers to the professional knowledge of an organization’s specialties, and is positively associated with organizational innovativeness [27]. Managers’ knowledge of nutrition influenced whether they perceived a need to improve the food environment in their facilities by adopting the ANGCY. The manager of the non-adopter facilities believed that foods that were homemade, fresh, and ‘real’ were healthy. As such, he saw no need to adopt the ANGCY because he considered the hamburgers and hot dogs made with 100% beef, homemade soups, hot chocolate made with fresh milk and most of the other items available in his concessions to be healthy. In keeping with the ANGCY, managers from adopter facilities understood nutritional quality to be a function of the micro and macronutrient content of foods. On this basis, they recognized that the majority of the foods available in their facilities were not healthy.

Professional knowledge was also highly important for ANGCY implementation, as in all cases of successful implementation Registered Dietitians assisted industry to reformulate menu items and/or to identify items that met the definition of “choose most often”.

*Size of operation, technical capacity:* Organizations that are larger, more mature, and that have greater technical resources tend to be more innovative [27]. Larger recreational facilities had a larger customer base and consequently their concessions had longer hours of operation. Their concessions also had more equipment, space for food storage and preparation and more highly skilled employees. These factors provided greater flexibility in their ability to prepare, store and sell healthier items and thus they could more easily adopt the ANGCY. Smaller concessions within single purpose facilities failed to adopt the ANGCY in part because they lacked this technical capacity.

*Absorptive capacity for new knowledge:* Recreational facilities that are able to identify and integrate new knowledge into their existing knowledge base will be better able to assimilate the ANGCY [27]. Prerequisites include the facility’s pre-existing technical infrastructure, formal expertise, organizational know-how and interpersonal networks [48]. There was limited pre-existing capacity to implement the ANGCY within all facilities, and therefore adopters sought to leverage their existing food service partnerships with industry in a health promoting direction. Several of these food vendors had already developed capacity to implement the ANGCY in schools and were willing to transfer this learning to the recreational facility setting. Their willingness to adopt the ANGCY and to be responsible for implementation was an important facilitator: “What things made it easier? I guess just the simple fact that we didn’t have to do any work… We didn’t have to go out there and do research to find out how much of what is in what and how big and how is it made and how much salt… Thank goodness we didn’t have to do that!”

By contrast, food vendors within the semi- and non-adopter facilities that did not agree to adopt the ANGCY either had no school-based operations, or were not using the ANGCY within their school-based operations. Thus, industry’s use of the ANGCY in schools built transferable capacity for implementing the ANGCY in recreational facilities. When health promoting partnerships with industry were not present, ANGCY adoption and implementation did not occur.

*Risk-taking climate:* A risk-taking climate was present within all facilities, however managers differed on the type of risks they were willing to take. Adopters were willing to accept the small financial risk of implementing the ANGCY in a choice-based format, but not the much greater risks inherent in a restrictive format. Conversely, the non-adopter had little tolerance for experimentation with initiatives that were not specifically intended to improve profitability, such as the ANGCY: “Part of the problem that we have is that we are under a great deal of pressure to meet our budget – like a great deal of pressure. So to experiment with things, it has to be something that we know is going to do well and is not going to end up costing us money or add on staffing hours.”

*Managerial relations:* Good relations between managers from recreational facilities and the food vendors operating within these facilities were present in all cases of ANGCY adoption. The juxtaposition of good and bad relationships within the semi-adopter was instructive. A good relationship between the facility and the vending machine company was the means by which the barrier posed by the company’s 20 year contract with the facility was overcome, whereas a poor relationship with one concession manager cemented this barrier in place and ultimately determined the status of the facility as a “semi-adopter”. Similarly, the good working relationship between the semi-adopter and the vending machine company supported ongoing implementation of the ANGCY despite the decline in revenues that ensued.

##### Organizational readiness for the ANGCY

*Fit of the ANGCY with the recreational facility context:* The ANGCY are more likely to be assimilated if they fit the recreational facilities’ existing values, norms, goals, skills, supporting technologies and ways of working [27]. Although nutrition was not a formal focus for the recreation sector, adopters sought to raise the priority of nutrition within their facilities by connecting the ANGCY to achievement of their organizational goals of supporting healthy lifestyles in the community. Because food services within non-adopter facilities were managed separately from the full facilities, they did not share the facilities’ overarching wellness goals and the priority of nutrition remained low. The ANGCY were a poor fit within this context.

*Tension for change:* For ANGCY adoption to occur, recreational facilities must perceive that their current food provision is not ideal, that the ANGCY can ameliorate the spread between their current and ideal food provision, and that change is an immediate imperative. The poor fit among adopters` personal beliefs, their organizational mandates and practices elevated tension for change and prompted organizational reform. By contrast, the manager for the non-adopter facilities was principally concerned with maintaining profitable food service operations, and perceived that ANGCY adoption might further compound existing financial stress.

##### Communication and influence

*Champion:* Champions are key individuals who are willing to throw their support behind an innovation and endeavour to overcome organizational indifference or resistance to a new idea [26]. Managers in adopter facilities “championed” the ANGCY within their facilities. Their qualities as champions were particularly evident when they requested that food vendors remove some highly profitable, but unhealthy items from the premises and when they remained committed to implementation despite declining revenues. The non-adopter recognized that not having an influential leader to champion the ANGCY was a barrier to adoption.

*Diffusion and dissemination of the ANGCY:* The influences available to help spread the ANGCY lie on a continuum from pure diffusion in which spread is largely unplanned, informal, and peer-mediated, to active dissemination, in which planned, formal programs and strategies are enacted to accelerate spread [27]. Adopters became aware of the ANGCY through formal dissemination channels, although information distributed in this way did not reach the non-adopter. Formal dissemination did not, however, provide a sufficient stimulus for ANGCY adoption by adopters. Instead, managers were motivated to seriously consider adoption when others within their social networks shared how they were using the ANGCY in schools, and encouraged them to do the same. No one had ever discussed ANGCY adoption with the non-adopter.

##### Outer context

*Competitive environment:* Competition was not a pressing issue for managers of adopter facilities. These facilities were the largest in their respective municipalities and there was little concern that patrons might frequent other facilities or bring in food from outside sources. Conversely, the opening of a new modern multiplex in the municipality where non-adopter facilities were located had the corollary effect of reducing facility and food service patronage. These competitive pressures created an uncertain environment that left little latitude for experimentation with menu items that might prove unprofitable, thereby discouraging ANGCY adoption.

*Interorganizational norm-setting:* The ANGCY are more likely to be adopted if a threshold proportion of organizations have adopted, or plan to adopt them [27]. According to managers, most patrons regarded recreational facilities as venues for unhealthy eating and therefore industry norms favoured unhealthy options. Adopters were willing to contravene these norms. The non-adopter indicated that he would have been more likely to adopt the ANGCY if “it became common that it was just that’s what facilities do… and if you went into a facility and they didn’t have, you know, fresh fruit or fresh vegetables, it would be kind of like that’s weird, sort of thing.” Thus, the current environment in which unhealthy foods were the norm discouraged him from adopting the ANGCY.

### Factors related to the implementation process

We conclude by describing factors related to the implementation process. These factors were similar among adopters, however comparison to non-adopter facilities was not possible because they had not implemented the ANGCY. We are therefore unable to judge to what extent these factors may have been sufficient and/or necessary for ANGCY implementation.

Managers perceived adoption as a simple matter, whereas they described implementation as much more challenging: “Adopting was as easy as writing a policy and now the work begins with trying to find people who are able to, you know, develop programming around that and really implement it properly.” Adopters expressed frustration with the unfinished state of implementation and its apparent ineffectiveness. Healthy options had always been available, implementation of the ANGCY simply meant there were now more of them. Meanwhile, unhealthy foods continued to dominate the food landscape. Notably, it was the manager of the full adopter facility who expressed the strongest sentiment in this regard: “I really think that we’ve missed the mark with implementing… It’s one thing to have it in paper and contracts but it’s another thing to deliver it… It’s disheartening to see what doesn’t happen. Like, it’s not as simple as writing a policy and people picking it up. It just doesn’t work that way.” Managers recognized that assimilation of the ANGCY within the organization’s systems and structures would take time: “Am I jumping up and down saying we did it? No, because there’s more to do. But at this point in time, in the short-term, we can’t see that changing. Maybe long-term… There’s a lot more we can do in terms of integrating [nutrition] within our services and programs. So that will come with time.”

Implementation ultimately depended on the leadership and direction of facility managers, and therefore when time limitations prevented them from focussing their attention on the ANGCY no progress was achieved: “Other priorities haven’t let me focus any energy here in quite some time… It just sits on the back burner. And so I’d say I’m a huge barrier, if you’re looking at barriers and facilitators… Nothing is actively happening… and there is no plan to do differently or roll out anything new.” Because of these time limitations managers attempted to devolve most of the responsibility for implementation to food vendors. Managers trusted them to implement the ANGCY, and did not monitor their progress in a formal and systematic manner. Instead, they gauged the status of implementation based on their own periodic observations and anecdotal reports from customers: “Probably every couple of weeks as I’m walking through the halls, I take note of what’s in there… but it’s not a checklist. It’s just, what do I see, what do I observe. If there’s something that catches my eye that’s kind of off, we’ll address it.” This lack of monitoring likely contributed to the ambivalence surrounding ANGCY implementation, as no one could be sure whether their efforts had been worthwhile.

### Propositions

The analysis culminated in the development of 25 propositions, presented in Table 7.

**Table 7:** **Propositions regarding factors from Greenhalgh et al’s Diffusion of Innovations framework**[27]**that were not common across all cases**

**Theoretical domain**	**Proposition**
**Food environment analysis**	Profit-oriented food services are incompatible with healthy environmental defaults (ie. > 50% CMO items), regardless of whether they are municipally or privately operated.
**Sales analysis**	Patrons insufficiently choose healthy options when the environmental defaults are unhealthy (ie. < 50% CMO items).
***Adopters and the adoption process***	
**Meaning of the ANGCY to managers**	1) Adoption and implementation of nutrition guidelines is greatest when the personal beliefs of managers, the organizational mandate and the aims of nutrition guidelines are all aligned.
	2) The personal beliefs of managers are highly influential and may motivate adoption when a window of opportunity arises.
***Attributes of the ANGCY***	
**Complexity**	Guidelines that are easily understood may be more readily adopted.
**Relative advantage**	1) Profitability is the most important barrier to adopting nutrition guidelines because managers perceive that selling healthy foods is unprofitable.
	2) A choice-based format may assist facilities to balance wellness and revenue concerns associated with nutrition guidelines, but may not support greater purchase of healthy items by patrons.
	3) Nutrition guidelines are perceived to provide a relative advantage insofar as they assist recreational facilities to achieve their wellness mandate in a financially sustainable manner. Small financial losses may be accepted if implementation supports achievement of other important priorities.
***Organizational antecedents for the ANGCY***	
**Formalization**	Short-term food service agreements provide greater flexibility to address emerging priorities.
**Professionalism**	1) Managers who correctly perceive their food environment as unhealthy are more likely to adopt nutrition guidelines.
	2) Registered Dietitians are a source of critical expertise to support implementation of nutrition guidelines.
**Size of operation, technical capacity**	Large recreational facilities may have greater technical capacity to implement the ANGCY.
**Absorptive capacity for new knowledge**	1) Use of nutrition guidelines in schools can create a favourable climate and increase capacity for adopting nutrition guidelines in other contexts.
	2) Health promoting partnerships with industry can provide capacity to implement nutrition guidelines that recreational facilities lack.
**Risk-taking climate**	Tolerance for financial risk is essential for adoption and implementation of nutrition guidelines.
**Managerial relations**	Where private industry is present, adoption and implementation of nutrition guidelines requires their full cooperation. When industry is committed to implementation, the stipulations of policies and contracts may be less important.
***Organizational readiness for the ANGCY***	
**Power balances**	Choice-based nutrition policies are better accepted by most stakeholders and may therefore facilitate adoption of nutrition guidelines.
**Fit of the ANGCY with the recreational facility context**	When food service is managed as a separate entity and is not under the direct purview of the general manager, its goals may not support adoption of nutrition guidelines.
**Tension for change**	Adoption of nutrition guidelines is more likely when management perceives a high tension for health-related change.
***Communication and influence***	
**Champion**	Managers act as gatekeepers of the food environment, and therefore an influential manager must champion adoption and implementation of nutrition guidelines.
**Diffusion and dissemination**	Use of nutrition guidelines in schools may facilitate spread to other contexts where diffusion networks are not yet active.
***Outer context***	
**Competitive environment**	Facilities that perceive fewer competitive pressures may be more likely to adopt nutrition guidelines.
**Interorganizational norm-setting**	1) Early adopters must be willing to accept the risks inherent in contravening industry norms.
	2) Diffusion of nutrition guidelines may be slow to occur because of the association of unhealthy foods with sport spectatorship.
***Implementation process***	The absence of clear goals and priorities for implementation and failure to monitor its progress can impede the implementation process.

ANGCY: Alberta Nutrition Guidelines for Children and Youth; CMO: “choose most often”.

### Long-term follow up

Six to 18 months following completion of each case study all facilities confirmed that their adoption status was stable, and that no major nutrition-related changes had been made to their food services.

## Discussion

### Food environment quality

Collectively, findings suggest that the food environment in facilities that have adopted and implemented the ANGCY may not be superior to the food environment in facilities that have not adopted the ANGCY. Although adopters made changes to their food environment, these changes were not substantial and did not create truly healthy food environments. These results are consistent with the only other published study of the impact of government nutrition guidelines on the food environment in recreational facilities. That study documented a 19% improvement in facility environment assessment scores and in availability of “choose most” and “choose sometimes” items in vending machines which, although statistically significant, nevertheless meant that the average post-intervention facility environment score was only 59% and only 17% of vending items were “choose most” [15]. That substantial voluntary change appears to be so difficult to achieve in recreational facility food services is perhaps unsurprising in light of current funding models, which make facilities partially dependent on the sale of unhealthy foods.

The availability of “choose most often” items was low within all of the facilities and was not consistently higher in adopter compared to non-adopter facilities. This outcome was partially a reflection of the low availability of such items in the marketplace, as for example, there are few “choose most often” food items suitable for sale within unrefrigerated vending machines. Although < 20% of items overall were “choose most often” in all facilities, ANGCY implementation and NEMS-R scores were often high, suggesting that policy makers and researchers should reconsider what constitutes a healthy food environment. Specific, high targets for the proportion of items that must fall within the “choose most often” category (ideally 100%) would help to ensure that nutrition guidelines support healthy food environments.

### Factors that influenced adoption and implementation of nutrition guidelines

This study systematically applied a Diffusion of Innovations framework to better understand adoption and implementation of nutrition guidelines in recreational facilities in Alberta. We assumed causal complexity, that is, that there are multiple paths to adoption, and thus we used a set theoretic approach to discern three sets of factors [43]: 1) Factors that were common to all cases and were therefore not sufficient to compel or dissuade adoption and implementation of nutrition guidelines. 2) Factors that were unique to individual cases and not consistently associated with adoption and implementation. These factors may be influential in particular contexts. 3) Factors that distinguished adopters from the non-adopter, and were therefore sufficient and perhaps also necessary for adoption and implementation. The specific paths by which adoption and implementation may occur are not known, however, as many different combinations of these factors are possible [49]. In addition, it would be premature to discard factors within the first category as unimportant, as although they do not guarantee adoption and implementation, they may nevertheless prove essential in future studies [43]. Our analysis suggests that it is primarily factors within the third and perhaps also the second categories that determine whether or not adoption and implementation of nutrition guidelines will occur within a given context.

Although the specific adoption trajectories differed among cases, several important findings emerged. First, the keys to adoption and implementation relate to the manager. The manager is a reflective decision-maker whose beliefs, perceptions, and knowledge shape his decisions and actions. Adoption and implementation of nutrition guidelines in recreational facilities is more likely when the manager personally values healthy eating, has a broad scope of responsibility encompassing wellness, regards nutrition guidelines in a positive light, perceives a high tension for health-related change within his facility’s food environment, is willing to champion changes that contravene industry norms and that may be financially risky, perceives few competitive pressures, maintains good relations with industry, and is willing to partner with them to achieve desired outcomes.

The fact that adopters were willing to eschew industry norms and adopt the ANGCY despite potential negative repercussions marks them as innovators [50]. These individuals are critical to diffusion as they act as gatekeepers, importing new ideas into a system [26]. Managers, however, do not have free reign. Their decisions are made within a particular micro and macroenvironmental context that is a source of facilitators and barriers. Barriers, including poor managerial relations, financial constraints, limited capacity to implement nutrition guidelines, unfavourable power balances, and the provisions of food service contracts impeded action on the part of managers. Financial constraints in particular, were a strong and consistent barrier to adopting and implementing the ANGCY in all facilities, as sales reductions caused managers to question the degree to which the ANGCY would provide them with an advantage relative to their previous practices. We were unable to objectively verify whether offering healthier foods was profitable in this context, and evidence from other studies in recreational facilities [15,24,51] and schools is conflicting [52-60] in this respect.

The challenge to balance support for affordable opportunities to be physically active with the need to promote healthy dietary behaviors is considerable in recreational facilities. Managers perceived that adopting the ANGCY in a choice-based format helped them to balance these competing priorities. Simply adding more healthy options to existing, largely unhealthy menus may not influence children’s dietary behaviors, however, as exhibited by students’ purchases in the full adopter facility. When given a choice, children tend to select unhealthy items [60-67]. Parents too, at times may make poor nutritional choices for their children because powerful social factors, time [68] and informational constraints [69] can easily take precedence over longer-term, intangible health concerns. Providing individuals with both healthy and unhealthy options (ie. a choice-based format) and trusting them to choose the healthiest option in spite of environmental conditions that overwhelmingly promote the opposite is unlikely to curtail escalating obesity rates.

The second major finding that emerged from this study is that although managers played a major role in adopting and implementing nutrition guidelines, they could not accomplish these tasks alone. Intersectoral linkages and formal health promoting partnerships were essential. Multisectoral, health promoting partnerships have long been recognized as a fundamental ingredient in effective health promotion practice [70]. It is difficult to envision how effective solutions to obesity can be forged without active involvement from the corporations that control and shape the food supply [71]. In the context of implementation of voluntary nutrition guidelines, adopters recognized that they lacked capacity to implement the ANGCY and therefore requested assistance from industry, leveraging their existing collaborative relationships in a new, health promoting direction. Where health promoting public-private partnerships existed, adoption and implementation proceeded, whereas no action was taken in their absence. The sustainability of these partnerships is unclear, however.

In addition to formal partnerships, informal linkages with schools were important. Adopters were motivated to seriously consider adopting the ANGCY when others within their social networks shared how they were using the ANGCY in schools, and encouraged them to do the same. Diffusion of the ANGCY therefore occurred within municipalities, from schools to recreational facilities, rather than among recreational facilities, as adoption of the ANGCY was too low for diffusion networks to become activated in this context [16]. In addition, industry’s willingness to collaborate with recreational facilities was partially determined by their pre-existing capacity to implement the ANGCY, developed through their school-based operations. Thus, efforts to improve the school food environment provided a supportive context and capacity to implement similar measures in recreational facilities.

Voluntary initiatives such as the ANGCY are of limited effectiveness in counteracting the pervasive influence of macro-level forces within the food system, as the environmental supports for voluntary action are poor. ANGCY uptake may therefore continue to falter under the current voluntary approach, and where it does occur, our findings suggest changes to the food environment may be relatively minor. Stronger government action is required to promote healthy dietary behaviors among children. Such action could include relatively less coercive (eg. incentives) or more coercive measures (eg. regulation). First and foremost, funding models should not be antithetical to recreational facilities’ wellness mandates. Facilities derived a small percentage of their overall revenues from food services and sponsorships, and thus it would not be costly to replace this revenue. Next, the ANGCY should be revised to include specific, measurable and robust recommendations. Other actions could include financial incentives (eg. tax breaks) for industry to develop products that meet the definition of “choose most often” and for those corporations that succeed in selling, not simply offering, a high proportion of “choose most often” items. Similar to pay-for-performance schemes in health care, governments could incorporate guideline-related outcomes as performance accountabilities for recreational facilities to continue to receive a portion of their public funding. Finally, governments could simply mandate that all recreational facilities adhere to the ANGCY, ideally in a restrictive format. Although some may argue that such measures interfere with the individual’s right to choose, many current policies already constrain food choice within recreational facilities (eg. funding models that make facilities partially dependent on food service revenues) and therefore such measures would merely counter existing obesogenic policies. These findings illustrate the tension that exists among individual rights, profitability and public health within market-based economies, and will assist policy makers to formulate policies that balance these competing interests.

### Strengths and limitations

This study was unique and had many strengths, including its in-depth nature and the range of cases studied. Mixed methods provided a more comprehensive understanding of the research questions than could have been achieved with a single approach. Multiple quantitative and qualitative perspectives of the food environment highlighted the many ways in which the food environment can be conceptualized, and showed that using a single tool is likely to yield incomplete and biased findings. We used a novel theoretical framework to discern factors that influenced uptake of the ANGCY, a model that may now provide a theoretical platform from which to investigate the uptake and operationalisation of a variety of obesity prevention policies. Finally, we contacted facilities 6–18 months following each case study to ascertain whether their adoption status had changed, and whether they had made any nutrition-related changes to food services.

ANGCY implementation scores were not consistently higher among adopters, and indeed, were high in some non-adopter facilities. This result was consistent with adopters’ perceptions that the food environment did not change substantially following ANGCY implementation. These results may also suggest a problem with the scoring system, as although the tool was judged to have good content validity, its construct validity may be poor. It is possible that the tool is not sensitive enough to differences between adopters and non-adopter facilities, as facilities could only receive a score of 0, 1 or 2 for each item. Others, however, have used similar scoring systems with good results [40]. Alternatively, the inability of the scoring system to distinguish adopter from non-adopter facilities may reflect problems within the ANGCY themselves, as informants felt several ANGCY recommendations were simply good business practice and likely to be practiced in all facilities. The guidelines also lack specific, measurable targets, which made it difficult to judge the degree to which facilities had implemented the recommendations. All of these factors likely contributed to the poor performance of the scoring system, however we believe the latter two were particularly influential.

We used multiple, mixed tools to assess food environment quality, however even these tools could not fully capture its many dimensions. We focussed mainly on physical aspects of the micro food environment, and did not extensively investigate its political, sociocultural and economic aspects [72], nor did we capture the subjective perceptions of patrons. Because we assessed the food environment at a single time point we could only infer change in food environment quality from managers’ comments and from comparison of adopter and non-adopter facilities. There is no universally agreed upon definition of a healthy food, and it is likely that a higher percentage of items would have been classified as healthy (ie. “choose most often”) using different standards, as ANGCY standards for sodium, in particular, are very stringent.

We make no claim that cases in this study are representative of all recreational facilities in Alberta. However, we have highlighted broad areas to target for change and provided as much detail as possible to allow the reader to evaluate the opportunities for generalization to other contexts.

## Conclusions

This study investigated factors influencing uptake of nutrition guidelines in recreational facilities in a real world context. Findings showed that when a voluntary system is in place, the keys to adoption and implementation of nutrition guidelines in recreational facilities relate to the manager’s nutrition-related knowledge, beliefs and perceptions, as these shape his decisions and actions. Policy dissemination strategies could therefore target these areas. The manager, however, cannot accomplish adoption and implementation alone. Intersectoral linkages with schools and formal health promoting partnerships with industry were also important for adoption and implementation to occur. Voluntary action and meaningful gains may, however, not be realized in an environment of long term food service contracts, limited support for change, funding models that depend on selling unhealthy food for profit, and relatively few palatable healthy products to substitute. Stronger nutrition guidelines and government support for product innovation may be needed.

Providing easy access to foods of poor nutritional quality in order to finance other social goods and preserve profitability is at odds with society’s ethical obligations to provide benefit and avoid harm to children [73]. Although some recreational facilities may argue that they cannot afford to lose revenue by implementing nutrition guidelines, the health and financial costs of not doing so may be much higher. Data from this study contribute to a better understanding of the factors that are maintaining many recreational facility food environments in an obesogenic state, and of the levers that can be used to tip them in more healthful directions.

## Abbreviations

ANGCY, Alberta Nutrition Guidelines for Children and Youth; CHO, carbohydrate; CMO, choose most often; kcals, calories; NEMS-R, Nutrition Environment Measures Survey in Restaurants.

## Competing interests

LJM received funding from the Government of Alberta to prepare a background literature review and draft version of the ANGCY. DLO and KDR were members of the committee that prepared the literature review and draft version of the ANGCY. The authors have no other competing interests.

## Authors’ contributions

DLO: designed the study, collected, analyzed and interpreted the data, wrote the manuscript. KDR: designed the study, interpreted the data, edited the manuscript. LJM: obtained funding for the study, designed the study, interpreted the data, edited the manuscript. All authors read and approved the final manuscript.

## Pre-publication history

The pre-publication history for this paper can be accessed here:

http://www.biomedcentral.com/1471-2458/12/376/prepub

## Supplementary Material

Additional file 1 ** Theoretically-informed, semi-structured interview guide.** Description of data: Questions were initially asked to open up areas of inquiry, and were followed by targeted probes when required. Theoretical domains addressed by each question are listed in brackets. The domains for the probes are the same as for the parent question except where otherwise indicated. Click here for file
