# Supplementary material for: Adopting and implementing nutrition guidelines in recreational facilities: Public and private sector roles. A multiple case study
Source: BMC Public Health. 2012 May 25;12:376. doi: 10.1186/1471-2458-12-376 (PMC3490726; doi:10.1186/1471-2458-12-376)
Supplement: Additional file 1 — Theoretically-informed, semi-structured interview guide. Description of data: Questions were initially asked to open up areas of inquiry, and were followed by targeted probes when required. Theoretical domains addressed by each question are listed in brackets. The domains for the probes are the same as for the parent question except where otherwise indicated. [file 1471-2458-12-376-S1.pdf]

Additional file 1

Theoretically-informed, semi-structured interview guide. Questions were initially asked to open up areas of inquiry, and were followed by targeted probes when required. Theoretical domains addressed by each question are listed in brackets. The domains for the probes are the same as for the parent question except where otherwise indicated.

1. What is the history of the facility and its food service?
2. Can you describe your role within the organization?
3. Tell me about when and how you first learned of the ANGCY. (Communication and influence)
4. How did you come to believe it was important for you to adopt and implement the ANGCY? (Communication and influence)
5. Who made the decision to adopt the ANGCY? (Adoption process)
    a. How was the decision made?
    b. When was the decision made?
    c. What reasons were given?
6. Thinking about adoption, which is a one-time mental decision to use the ANGCY, what were the barriers to adopting the ANGCY? (Adoption process)
    d. Which were the most important?
    e. How did you address these barriers? How could these barriers to overcome?
7. What things made it easier to adopt the ANGCY? (Adoption process)
    f. What was the most important factor?
    g. What things would have made it easier to adopt the ANGCY?
8. How did you go about developing nutrition policies? (Adoption process)
9. Thinking about implementing the ANGCY, which are the concrete steps to put the ANGCY into practice, what are all the things you had to do to implement the ANGCY? (Implementation process)
    h. Which aspects of the ANGCY did you implement and why?
        i. What changes did you make to the ANGCY during implementation? (Attributes of the innovation)
        ii. Did you implement any of your own nutrition policies/programs that were not part of the ANGCY? (Attributes of the innovation)
        iii. What aspects of the ANGCY did you want to implement but were unable to? (Organizational readiness for innovation)
    i. What resources did you have already and what resources did you have to acquire to be able to implement the ANGCY? (Organizational antecedents for innovation)
    j. How did you communicate changes to your staff, and how did they provide you with feedback on the changes? (Communication and influence)
    k. What was the timeline?
    l. What do you define as successful implementation?
10. What were the barriers to implementing the ANGCY? (Implementation process)
    m. Which were the most important?
    n. How did you address the barriers to implementation?
11. What things made it easier to implement the ANGCY? (Implementation process)
    o. Which were the most important?

p. What things would have made it easier to implement the ANGCY?

12. What factors in the wider environment helped/hindered adoption and implementation? (Outer context)
13. Can you describe the internal and external support and opposition you had throughout adoption and implementation? (Organizational readiness for innovation)
14. In what ways did the ANGCY fit/not fit with your values and priorities? (Organizational readiness for innovation)
15. In what ways did the ANGCY fit/not fit with your ways of working and skill sets? (Organizational readiness for innovation)
    q. What changes did you make to your ways of working to better fit the ANGCY?
16. How do you make sure the ANGCY continue to be followed? (Processes of assimilation and implementation)
17. What outcomes have you observed from implementing the ANGCY? (Attributes of the innovation)
18. As you look back on adopting and implementing the ANGCY, are there any other important factors that stand out in your mind? (Assimilation process)
19. What advice would you give to a recreation facility manager who is deciding whether to adopt/implement the ANGCY? (Assimilation process)
    r. What, if anything would you do differently next time?
20. What are your future plans with respect to the ANGCY? (Assimilation process)
